# Supplementary material for: Modeling glioblastoma heterogeneity as a dynamic network of cell states
Source: Mol Syst Biol. 2021 Sep 16;17(9):e10105. doi: 10.15252/msb.202010105 (PMC8444284; doi:10.15252/msb.202010105)
Supplement: Supplementary file 6 — Source Data for Figure 5 [file MSB-17-e10105-s004.zip › Figure5A_sourcedata/GSEA_3071/hallmarks_stateVI.GseaPreranked.1623742557031/HALLMARK_TNFA_SIGNALING_VIA_NFKB.html]

Details for gene set HALLMARK\_TNFA\_SIGNALING\_VIA\_NFKB[GSEA]

|  || Dataset | state63071 |
| Phenotype | NoPhenotypeAvailable |
| Upregulated in class | na\_pos |
| GeneSet | HALLMARK\_TNFA\_SIGNALING\_VIA\_NFKB |
| Enrichment Score (ES) | 0.35451335 |
| Normalized Enrichment Score (NES) | 2.4934328 |
| Nominal p-value | 0.0 |
| FDR q-value | 0.0 |
| FWER p-Value | 0.0 |
Table: GSEA Results Summary

  

Fig 1: Enrichment plot: HALLMARK\_TNFA\_SIGNALING\_VIA\_NFKB      
 Profile of the Running ES Score & Positions of GeneSet Members on the Rank Ordered List

  

| PROBE | GENE SYMBOL | GENE\_TITLE | RANK IN GENE LIST | RANK METRIC SCORE | RUNNING ES | CORE ENRICHMENT || 1 | SERPINE1 |  |  | 0 | 1.673 | 0.0337 | Yes |
| 2 | F3 |  |  | 26 | 0.988 | 0.0465 | Yes |
| 3 | PLAUR |  |  | 34 | 0.936 | 0.0634 | Yes |
| 4 | EDN1 |  |  | 50 | 0.866 | 0.0765 | Yes |
| 5 | ETS2 |  |  | 52 | 0.864 | 0.0936 | Yes |
| 6 | PTX3 |  |  | 56 | 0.860 | 0.1101 | Yes |
| 7 | MYC |  |  | 69 | 0.815 | 0.1231 | Yes |
| 8 | INHBA |  |  | 80 | 0.781 | 0.1360 | Yes |
| 9 | GADD45B |  |  | 90 | 0.758 | 0.1487 | Yes |
| 10 | PPP1R15A |  |  | 98 | 0.738 | 0.1616 | Yes |
| 11 | PHLDA2 |  |  | 126 | 0.697 | 0.1680 | Yes |
| 12 | DUSP1 |  |  | 136 | 0.685 | 0.1793 | Yes |
| 13 | SPHK1 |  |  | 141 | 0.677 | 0.1918 | Yes |
| 14 | SQSTM1 |  |  | 147 | 0.667 | 0.2038 | Yes |
| 15 | DUSP5 |  |  | 169 | 0.631 | 0.2106 | Yes |
| 16 | HBEGF |  |  | 172 | 0.627 | 0.2226 | Yes |
| 17 | TNIP1 |  |  | 183 | 0.618 | 0.2322 | Yes |
| 18 | BIRC3 |  |  | 191 | 0.608 | 0.2425 | Yes |
| 19 | GADD45A |  |  | 200 | 0.603 | 0.2524 | Yes |
| 20 | GEM |  |  | 233 | 0.577 | 0.2550 | Yes |
| 21 | IL18 |  |  | 239 | 0.572 | 0.2651 | Yes |
| 22 | TNFAIP2 |  |  | 244 | 0.568 | 0.2754 | Yes |
| 23 | SPSB1 |  |  | 248 | 0.566 | 0.2859 | Yes |
| 24 | MAFF |  |  | 253 | 0.562 | 0.2961 | Yes |
| 25 | RIPK2 |  |  | 267 | 0.555 | 0.3036 | Yes |
| 26 | JUN |  |  | 321 | 0.527 | 0.2993 | Yes |
| 27 | FOSL1 |  |  | 339 | 0.516 | 0.3049 | Yes |
| 28 | CXCL3 |  |  | 359 | 0.508 | 0.3097 | Yes |
| 29 | PLK2 |  |  | 363 | 0.505 | 0.3191 | Yes |
| 30 | RELB |  |  | 406 | 0.486 | 0.3170 | Yes |
| 31 | ACKR3 |  |  | 408 | 0.486 | 0.3265 | Yes |
| 32 | ATF3 |  |  | 416 | 0.483 | 0.3343 | Yes |
| 33 | GFPT2 |  |  | 436 | 0.476 | 0.3385 | Yes |
| 34 | TNFAIP3 |  |  | 445 | 0.473 | 0.3457 | Yes |
| 35 | EHD1 |  |  | 455 | 0.469 | 0.3526 | Yes |
| 36 | KLF10 |  |  | 506 | 0.453 | 0.3477 | Yes |
| 37 | MAP2K3 |  |  | 550 | 0.440 | 0.3444 | Yes |
| 38 | EIF1 |  |  | 568 | 0.434 | 0.3484 | Yes |
| 39 | DNAJB4 |  |  | 578 | 0.431 | 0.3545 | Yes |
| 40 | YRDC |  |  | 674 | 0.405 | 0.3359 | No |
| 41 | FOSL2 |  |  | 717 | 0.393 | 0.3320 | No |
| 42 | IL7R |  |  | 718 | 0.392 | 0.3399 | No |
| 43 | IRF1 |  |  | 771 | 0.382 | 0.3329 | No |
| 44 | SOCS3 |  |  | 775 | 0.381 | 0.3398 | No |
| 45 | KLF2 |  |  | 814 | 0.374 | 0.3366 | No |
| 46 | KLF6 |  |  | 854 | 0.368 | 0.3330 | No |
| 47 | JAG1 |  |  | 876 | 0.365 | 0.3344 | No |
| 48 | BMP2 |  |  | 940 | 0.354 | 0.3238 | No |
| 49 | IER5 |  |  | 950 | 0.353 | 0.3284 | No |
| 50 | NFIL3 |  |  | 956 | 0.352 | 0.3341 | No |
| 51 | SDC4 |  |  | 1013 | 0.343 | 0.3252 | No |
| 52 | TRAF1 |  |  | 1040 | 0.339 | 0.3247 | No |
| 53 | PDLIM5 |  |  | 1062 | 0.335 | 0.3255 | No |
| 54 | CEBPB |  |  | 1389 | 0.293 | 0.2396 | No |
| 55 | TNIP2 |  |  | 1401 | 0.292 | 0.2424 | No |
| 56 | TRIP10 |  |  | 1422 | 0.289 | 0.2426 | No |
| 57 | PANX1 |  |  | 1512 | 0.278 | 0.2231 | No |
| 58 | CLCF1 |  |  | 1592 | 0.269 | 0.2063 | No |
| 59 | RELA |  |  | 1791 | 0.252 | 0.1556 | No |
| 60 | SERPINB8 |  |  | 1800 | 0.251 | 0.1584 | No |
| 61 | LIF |  |  | 1804 | 0.250 | 0.1626 | No |
| 62 | ZBTB10 |  |  | 1941 | -0.270 | 0.1297 | No |
| 63 | SAT1 |  |  | 1952 | -0.271 | 0.1324 | No |
| 64 | SNN |  |  | 2012 | -0.281 | 0.1214 | No |
| 65 | VEGFA |  |  | 2104 | -0.298 | 0.1018 | No |
| 66 | TSC22D1 |  |  | 2193 | -0.313 | 0.0833 | No |
| 67 | DENND5A |  |  | 2332 | -0.339 | 0.0513 | No |
| 68 | NFE2L2 |  |  | 2707 | -0.427 | -0.0454 | No |
| 69 | CFLAR |  |  | 2902 | -0.495 | -0.0901 | No |
| 70 | PDE4B |  |  | 2948 | -0.513 | -0.0924 | No |
| 71 | LAMB3 |  |  | 2980 | -0.529 | -0.0905 | No |
| 72 | DUSP4 |  |  | 3007 | -0.545 | -0.0869 | No |
| 73 | ID2 |  |  | 3026 | -0.554 | -0.0808 | No |
| 74 | PNRC1 |  |  | 3055 | -0.566 | -0.0773 | No |
| 75 | TNFAIP6 |  |  | 3085 | -0.578 | -0.0738 | No |
| 76 | B4GALT5 |  |  | 3115 | -0.595 | -0.0700 | No |
| 77 | BTG3 |  |  | 3184 | -0.647 | -0.0761 | No |
| 78 | EGR1 |  |  | 3285 | -0.742 | -0.0894 | No |
| 79 | PMEPA1 |  |  | 3338 | -0.804 | -0.0879 | No |
| 80 | BTG2 |  |  | 3377 | -0.857 | -0.0813 | No |
| 81 | TGIF1 |  |  | 3380 | -0.860 | -0.0646 | No |
| 82 | PLPP3 |  |  | 3394 | -0.880 | -0.0505 | No |
| 83 | FJX1 |  |  | 3437 | -0.976 | -0.0427 | No |
| 84 | FOS |  |  | 3476 | -1.056 | -0.0322 | No |
| 85 | LITAF |  |  | 3491 | -1.089 | -0.0142 | No |
| 86 | NINJ1 |  |  | 3500 | -1.117 | 0.0060 | No |
| 87 | TNC |  |  | 3586 | -1.618 | 0.0146 | No |
Table: GSEA details [plain text format]

  

Fig 2: HALLMARK\_TNFA\_SIGNALING\_VIA\_NFKB: Random ES distribution      
 Gene set null distribution of ES for **HALLMARK\_TNFA\_SIGNALING\_VIA\_NFKB**
